# Comparative Evaluation of Salivary Sialic Acid Levels Among Beedi Rollers and Tobacco Users in Mangalore, South India

**DOI:** 10.7759/cureus.16651

**Published:** 2021-07-26

**Authors:** RJ Ancy, Rekha P Shenoy, Praveen S Jodalli, Laxminarayan Sonde, Imran P Mohammed

**Affiliations:** 1 Public Health Dentistry, Century International Institute of Dental Science and Research Centre, Kasaragod, IND; 2 Public Health Dentistry, Yenepoya Dental College and Hospital, Mangalore, IND

**Keywords:** smoking tobacco, smokeless tobacco, beedi rollers, cancer, salivary sialic acid, tobacco

## Abstract

Introduction: According to World Health Organization, the global cancer burden is estimated to have risen to 18.1 million new cases and 9.6 million deaths in 2018. Tobacco use is a leading cause of cancer and of death from cancer. Beedis are the most popular smoking form of tobacco in India. Thirty-four percent of the tobacco produced in India is used for making beedis. The beedi sector is agroforestry-based and the second largest industry in India with approximately 4.4 million full-time beedi workers in India. Toxic constituents present in tobacco are released into the ambient air during the processing of beedis.

Methods: A descriptive cross-sectional study was conducted to assess and compare salivary sialic acid levels among beedi rollers, tobacco smokers, smokeless tobacco users and individuals with no tobacco exposure. The study sample comprised of 140 individuals who were 30 to 60 years old, who attended dental screening and treatment camps in rural and urban areas in Mangalore, conducted by the Department of Public Health Dentistry, Yenepoya Dental College and patients who visited the Department of Oral Medicine and Radiology of Yenepoya Dental College. Saliva was collected by passive drool method into a sterile container. Biochemical analysis of salivary sialic acid was done using the acidic ninhydrin method. Continuous variables are expressed in terms of mean and standard deviation. Categorical variables are expressed in terms of frequencies and percentages. To compare salivary sialic acid levels between the groups ANOVA was used. The Chi-square test was used to compare categorical variables between the groups.

Results: A total of 140 participants, 35 beedi rollers, 35 smokers, 35 smokeless tobacco users and 35 individuals with no tobacco exposure participated in the study. Among the 140 participants, 90 participants were males and 50 participants were females. There was a statistically significant difference in the mean salivary sialic acid level between the different groups (p<0.001) with smokeless tobacco users having the highest (10.60 mg/dL) mean salivary sialic acid level. It was found that the mean salivary sialic acid level reduced as the age progressed, even though there was no statistically significant difference. There was a statistically significant difference in the mean salivary sialic acid level between the different groups (p=0.010) with participants with 11 to 20 years of exposure to tobacco having the highest (8.67 mg/dL) mean salivary sialic acid level and participants with no tobacco exposure having least (3.06 mg/dL) mean salivary sialic acid level.

Conclusion: The salivary sialic acid level was more in beedi rollers than individuals with no tobacco exposure, even though the difference was not statistically significant. The results showed elevated levels of salivary sialic acid in smokeless tobacco users followed by tobacco smokers. This may be an indication that smokeless tobacco use has harmful eﬀects similar to or more than tobacco smoking since salivary sialic acid levels in smokeless tobacco users were higher than those in smokers.

## Introduction

According to WHO data, it is estimated that the global cancer burden has risen to 19.3 million new cases and 10.0 million fatalities in 2020 [1].

The tobacco epidemic is one of the biggest public health challenges the world has ever faced killing more than eight million people around the world each year. More than seven million of those deaths are the result of direct tobacco use while around 1.2 million are the result of non-smokers being exposed to second-hand smoke [2]. There is a large number of carcinogens that exist in both smoked tobacco as well as smokeless tobacco [3,4].

In India, beedi consumption outpaces conventional cigarettes [5]. As beedis are a less expensive form of tobacco consumption, it is extremely popular among the poor. But it carries greater risks to health as it delivers more nicotine, carbon monoxide and tar than conventional cigarettes [6].

The Beedi sector is based on agroforestry and it is India's second-largest industry with approximately 4.4 million full-time beedi workers, most of whom are women who belong to lower socioeconomic status. The number of beedi workers in the state of Karnataka is approximately 3.6 lakhs, of which 2.5 lakhs are in the Mangalore region alone and of them, 80% are home-based beedi workers [7].

Toxic constituents present in tobacco are released into the ambient air during the processing of beedis. The nicotine concentration in the tobacco of beedi (21.2 mg/g) is significantly higher than that of commercial filtered (16.3mg/g) and unfiltered cigarettes (13.5 mg/g). The nicotine which is released from the tobacco leaves can be absorbed through the skin, respiratory epithelium, and mucous membrane of the mouth. Increased levels of tobacco constituents were found in the urine of beedi workers, which suggests increased systemic exposure to tobacco [8]. A study conducted by Bhisey et al. in 1991 at Bombay's Cancer Research Institute revealed that workers engaged in the manufacturing of beedis were exposed to dangerously high levels of carcinogens that are either absorbed through the skin or inhaled as dust [9]. 

Only a few studies have been done so far regarding the inhalation of unburnt tobacco dust as a risk factor for carcinoma. One such occupation where such a form of risk is involved is beedi rolling, which is a century-old business in India. In their work environment, beedi rollers handle tobacco flakes and inhale tobacco dust as well as volatile tobacco components that put them at a high risk of cancer [7].

Yasmin et al. in 2010 studied the health problems of female beedi rollers in Patna and found that more than 70% of the beedi rollers suffered from the eye, gastrointestinal, nervous, respiratory and osteological problems. This reveals that beedi rolling may cause signiﬁcant health hazards and beedi workers are at an increased risk for many health problems [10].

During tumor development, substances that change quantitatively in the serum are collectively referred to as tumor markers or biochemical serum markers. A marker is typically synthesized by the tumor and released into circulation or expressed in large amounts by malignant cells on the cell surface, then by their counterparts. Knowing the dynamic biology of cancer and using accurate biomarkers for the diagnosis and staging of malignant diseases and the assessment of different therapeutic methods will promote early detection, successful cancer treatment and prognosis, and can thus significantly improve survival [11,12].

Increased levels of serum and salivary sialic acid in oral cancer patients have been documented by various researchers. Sialic acids are sugar compounds at the end of glycoprotein and glycolipid oligosaccharide chains that play a significant role in the development of a neoplastic activity, as important cell membrane compounds [13,14]. Increased sialic acid is usually found in tumor tissues and is supposed to be a potential hallmark of cancer [15].

The alterations in glycoproteins start at an early stage of tumorigenesis. Gatchev et al. in 1993 presented first data on sialic acid levels before a diagnosis of tumor and reported elevated levels of sialic acid among men in whom a malignant tumor was diagnosed almost eight years after the estimation of sialic acid level [16].

During malignant transformation, multiple carcinogens present in tobacco may have a role in various biochemical and molecular changes. If these alterations can be identified well before any cancer-related physical changes, the chances of cancer prevention can increase. Studies have shown that sialic acid levels are elevated in smoke and smokeless tobacco users. Due to the carcinogenic effect of tobacco, measurement of these parameters in saliva may be useful as a risk marker of cancer [17].

Due to the non-invasiveness of its collection, the non-necessity of the skilled technicians for collection and the presence of sensitive biomarkers, saliva is considered an effective diagnostic tool. In addition, no previous studies have been done to assess sialic acid levels in beedi rollers. Therefore this study was conducted to assess and compare salivary sialic acid levels among beedi rollers, tobacco smokers, smokeless tobacco users and individuals with no tobacco exposure. Also, this study will help in drawing attention to the potential health hazards among beedi rollers, tobacco smokers and smokeless tobacco users. This will also help in timely screening and implementing protective measures to reduce exposure to tobacco in beedi rollers, and thereby prevent occupational hazards related to beedi manufacturing.

## Materials and methods

Study design

A descriptive cross-sectional study was conducted from January 2020 to February 2020 to assess and compare salivary sialic acid levels among beedi rollers, tobacco smokers, smokeless tobacco users and individuals with no tobacco exposure.

Study setting

The study sample comprised 30 to 60 years old adults, who attended dental screening and treatment camps in rural and urban areas in Mangalore, conducted by the Department of Public Health Dentistry, Yenepoya Dental College and patients who visited the Department of Oral Medicine and Radiology of Yenepoya Dental College. The study sample comprised of four groups:

 Group I: beedi rollers 

 Group II: tobacco smokers

 Group III: smokeless tobacco users

 Group IV: individuals with no tobacco exposure

Sample size

The estimated sample size was 35 in each group with a level of significance 5% and power 80% based on a previous study [8].

Sampling method

Most of the beedi rollers are home-based workers. So beedi rollers are unknown and difficult to find unless a member among them provides contact details of the other members. So in order to identify subsequent beedi rollers, the snowball sampling method was used for group I till the desired sample size was achieved. And convenience sampling method was used for group II, group III and group IV as they were easy to obtain.

Inclusion criteria

Individuals aged between 30 and 60 years who consented to participate in the study were grouped as:

- Group I: Individuals who are beedi rollers at least since 10 years who are not smokers or smokeless tobacco users and who do not have exposure to secondhand smoke at home or workplace.

- Group II: Individuals who are tobacco smokers at least since 10 years and who do not use smokeless tobacco.

- Group III: Individuals who are smokeless tobacco users at least since 10 years and are not tobacco smokers.

- Group IV: Individuals who were not exposed to tobacco at least 30 days prior to the examination.

Exclusion criteria

Uncooperative individuals or individuals who did not consent to participate in the study, individuals with systemic diseases like cardiovascular disease, hypertension, diabetes mellitus, asthma, etc., which influence sialic acid levels in the body, and individuals who were diagnosed with premalignant disorders and malignancy were excluded from the study. Since periodontitis is a confounding factor in the estimation of sialic acid, individuals with periodontitis (i.e., Community Periodontal Index [CPI] score 3 or above in any of the sextants) were also excluded from the study. This was done for the purpose of matching so that there will be equal distribution of the confounder.

Data collection

Ethical clearance was obtained from Yenepoya University Ethics Committee. Informed consent was obtained from the study participants prior to the start of the study. Participants were informed about the study protocol and were given adequate time to decide on participation in the study. They were informed that they can withdraw from the study at any time during the study. The demographic details such as age, gender, education, occupation, family income, socioeconomic status, duration of exposure to tobacco and brief medical history of the participants were recorded.

The periodontal health was assessed by using CPI. CPI score ranges from 0 to 4 and describes the periodontal condition of individuals at the population level. CPI score 0 indicates no periodontal disease. Score 1 indicates gingival bleeding on probing, score 2 indicates the presence of calculus and bleeding, score 3 indicates shallow periodontal pockets of 4-5 mm and score 4 indicates deep periodontal pockets of 6 mm or greater.

Clinical examination was performed using a plane mouth mirror and CPI probe under adequate natural light. An examination of the oral mucosa and soft tissues in and around the mouth was made on every participant for the presence of any oral mucosal lesions based on the WHO Oral Health Assessment Form for Adults 2013 [18].

Saliva was collected by the passive drool method. The passive drool method is a saliva collection method for collecting whole saliva (also called mixed saliva). In this method, saliva is allowed to pool at the bottom of the mouth and then individuals are asked to collect the pooled saliva into a sterile container. Biochemical analysis of salivary sialic acid was done using the acidic ninhydrin method by Gaitonde [19]. In the presence of an acidic medium, sialic acid reacts with ninhydrin to form a colored substance that can be spectrophotometrically measured at 470 nm. In this method, 0.1 mL of glacial acetic acid and 0.1 mL of acid ninhydrin reagent were added to 0.1 mL of a saliva sample. In a boiling water pan, the reaction mixture was heated for 10 min. After cooling the mixture under tap water, 1.0 mL of 99% of ethanol was added to the mixture. Absorbance was measured at 470 nm using a spectrophotometer.

Statistical analysis

The data collected were entered in Microsoft Excel Software and was exported to SPSS (Statistical package for social science) Version 23 (IBM SPSS Statistics Inc., Chicago, IL, USA) for the statistical analysis. The salivary sialic acid level which is a continuous variable is expressed in terms of mean and standard deviation. Categorical variables such as gender, age, socioeconomic status, and years of tobacco exposure are expressed in terms of frequencies and percentages. To compare salivary sialic acid levels between the groups, a t-test and ANOVA were used. And post hoc analysis (Bonferroni) was done. The level of significance was set at p < 0.05.

## Results

A total of 140 participants, 35 beedi rollers, 35 tobacco smokers, 35 smokeless tobacco users and 35 individuals with no tobacco exposure participated in the study. Sixty-four percent of the participants were males and 36% of participants were females. Among the participants, 51 participants were in the age group of 30-40 years, 37 participants were in the age group of 41- 50 years and 52 participants were in the age group of 51-60 years. And among the participants, 11 participants were from an upper middle class, 20 participants were from the lower middle class, 101 participants were from the upper lower class and 8 participants were from the lower class (according to Kuppuswamy socioeconomic status scale 2017).

The mean salivary sialic acid was estimated among the different groups. A one-way ANOVA test was used to compare the mean salivary sialic acid between the groups. There was a statistically significant difference in the mean salivary sialic acid level between the different groups (p<0.001) with smokeless tobacco users having the highest (10.60 mg/dL) mean salivary sialic acid level and individuals with no tobacco exposure having least (3.06 mg/dL) mean salivary sialic acid level (Table 1).

**Table 1:** Mean salivary sialic acid levels among different groups

Group	N	Mean	Standard Deviation	F	P-value
Salivary sialic acid (mg/dL)					
Beedi rollers	35	3.26	5.00	8.775	<0.001
Smokers	35	8.52	9.21		
Smokeless tobacco users	35	10.60	9.13		
Individuals with no tobacco exposure	35	3.06	6.00		

The paired comparison of mean salivary sialic acid levels among different groups was done using post hoc analysis (Bonferroni), which is shown in Table 2. There was a statistically significant difference in the mean salivary sialic acid level between beedi rollers and tobacco smokers (p=0.026), beedi rollers and smokeless tobacco users (p=0.001), tobacco smokers and individuals with no tobacco exposure (p=0.018) and smokeless tobacco users and individuals with no tobacco exposure (p<0.001).

**Table 2:** A paired comparison of mean salivary sialic acid levels among different groups

Paired Groups		Mean Difference	Standard Error	P-value
Salivary sialic acid				
Beedi rollers	Smokers	-5.26	1.81	0.026
Beedi rollers	Smokeless tobacco users	-7.34	1.81	0.001
Beedi rollers	Individuals with no tobacco exposure	0.20	1.81	1.000
Smokers	Smokeless tobacco users	-2.08	1.81	1.000
Smokers	Individuals with no tobacco exposure	5.46	1.81	0.018
Smokeless tobacco users	Individuals with no tobacco exposure	7.53	1.81	<0.001

The mean salivary sialic acid levels among different gender were compared. An independent t-test was used to compare the mean salivary sialic acid between the groups. There was a statistically significant difference in the mean salivary sialic acid level between the different groups (p=0.025) with males (7.88 ± 8.78 mg/dL) having higher mean salivary sialic acid levels than females (3.62 ± 6.15 mg/dL) (Table 3).

**Table 3:** Mean salivary sialic acid levels among different gender

Group	N	Mean	Standard Deviation	t	P-value
Salivary sialic acid (mg/dL)					
Male	90	7.88	8.78	5.124	0.025^*^
Female	50	3.62	6.15		

Table 4 shows the mean salivary sialic acid levels among different age groups. A one-way ANOVA test was used to compare mean salivary sialic acid between the groups. It was found that the mean salivary sialic acid level reduced as the age progressed, even though there was no statistically significant difference.

**Table 4:** Mean salivary sialic acid levels among different age groups

Group	N	Mean	Standard Deviation	F	P-value
Salivary sialic acid (mg/dL)					
30-40 years	51	8.13	9.39	2.131	0.123
41-50 years	37	6.01	6.92		
51-60 years	52	4.87	7.52		

Table 5 shows the mean salivary sialic acid levels among different groups based on years of exposure to tobacco. A one-way ANOVA test was used to compare the mean salivary sialic acid between the groups. There was a statistically significant difference in the mean salivary sialic acid level between the different groups (p=0.010) with participants with 11 to 20 years of exposure to tobacco having the highest (8.67 mg/dL) mean salivary sialic acid level and participants with no tobacco exposure having least (3.06 mg/dL) mean salivary sialic acid level.

**Table 5:** Mean salivary sialic acid levels among different groups based on years of exposure to tobacco

Group	N	Mean	Standard Deviation	F	P-value
Salivary sialic acid (mg/dL)					
No exposure	35	3.06	6.00	3.953	0.010^*^
10 years	20	8.02	6.21		
11-20 years	51	8.67	9.78		
More than 20 years	34	5.31	7.42		

## Discussion

Cancer is a life-threatening disease. Early detection accompanied by adequate care, can raise cure rates to 80% or 90%, which can dramatically boost the quality of life by minimizing prolonged, debilitating treatment procedures. Malignant cell studies have revealed changes to the sialic acid content of glycoproteins and glycolipids in cell surfaces and membranes. Sialic acid has been found to be released into the circulation during tumor progression.

In previous studies, the importance of serum sialic acid as a sensitive tumor marker has been identified. Due to the factors such as being inexpensive, non-invasive (basically an ultrafiltrate of blood), and simple to handle, saliva wins over blood as a diagnostic fluid to control health and disease. For patients, the non-invasive collection technique dramatically reduces anxiety, discomfort and simplifies the procurement of samples [20].

Thus, we estimated the salivary sialic acid levels in beedi rollers, tobacco smokers, smokeless tobacco users and individuals with no tobacco exposure.

In the present study, it was found that a large proportion of the women involved in beedi rolling are in this profession for more than 20 years, suggesting they start their profession at an early age. This finding was similar to a study conducted by Yasmin et al. in 2010 [10].

The salivary sialic acid level was higher in beedi rollers than in individuals with no tobacco exposure but was not statistically significant. And the salivary sialic acid level was lower in beedi rollers when compared to tobacco smokers and smokeless tobacco users. No studies are reported in the literature estimating salivary sialic acid levels in beedi rollers, and hence the results of our study cannot be compared with any other studies.

Increased serum and salivary levels of sialic acid have also been documented in patients with oral cancer. Because increased sialic acid in tumor tissues is found to be a potential hallmark of cancer. An increased concentration of sialic acids in tumor cells, and its secretion by some of these cells, increases its concentration in the blood or saliva. Therefore, the assessment of the level of glycoconjugates can be useful in the early diagnosis and staging of oral cancer, which is often related to the consumption of smokeless tobacco and cigarettes or beedis [13].

In the present study, the mean salivary sialic acid levels were found to be elevated in smokeless tobacco users (10.60 ± 9.13 mg/dL) as compared to tobacco smokers (8.52 ± 9.21 mg/dL) followed by beedi rollers (3.26 ± 5.00 mg/dL) and individuals with no tobacco exposure (3.06 ± 6.00 mg/dL). These findings were similar to the studies conducted by Mollashahi et al. in 2016 and Kurtul et al. in 2012 in which the salivary sialic acid levels were significantly higher in the smokeless tobacco users (paan, maras powder) (39.57 ± 26.58 mg/L, 75.52 ± 6.86 μg/mL) and smokers (62.60 ± 3.91 μg/mL) than individuals with no tobacco exposure (38.39 ± 28.55 mg/L, 51.60 ± 3.51 μg/mL) [13,17].

Taking into consideration the studies mentioned above, increased salivary sialic acid levels in smokers and smokeless tobacco users might be related to various diseases, for example, various cancers and cardiovascular disease. Cardiovascular diseases are mainly caused by atherosclerosis and sialic acid has a role in the pathogenesis of atherosclerosis. Previous studies have shown that sialic acid levels are elevated in cardiovascular disease and also in people with tobacco habits [13]. So cardiovascular disease can act as a confounder in the estimation of tobacco habits, due to which individuals with cardiovascular disease and other systemic diseases were excluded from the study. The increased level of salivary sialic acid in smokers and smokeless tobacco users may be a reflector of the risk of cardiovascular diseases.

The present study showed that the mean salivary sialic acid level was higher in males compared to females. This might be because of the gender difference in overall tobacco use. In general, men are more likely to use more tobacco products than women [21]. It was also noted that the mean salivary sialic acid level decreased as the age of the individuals progress. A study conducted by Jawzaly et al. in 2013 also showed that there is an inverse relation of age with sialic acid level. This might be due to the fact that immune response will decline with the age of the individuals [22]. Also, the mean salivary sialic acid level was found to be higher in the lower socioeconomic groups compared to the higher socioeconomic groups.

In the present study, it was observed that the mean salivary sialic acid level increased as the years of exposure to tobacco increased. But it showed a reduction in the mean salivary sialic acid level as the years of tobacco exposure progressed to more than 20 years. In contrary to this, studies conducted by Mollashahi et al. and Kurtul et al. found that mean salivary sialic acid level increased with an increased duration of tobacco exposure [13,17]. This might be due to the difference in the age group of the participants in our study compared to other studies, as there is a reduction in sialic acid level as the age progressed.

The limitation of this study included convenience sampling which can lead to under-representation or over-representation of particular groups within the sample. This can be avoided by ensuring that the samples are representative and well distributed so that the sample will have adequate diversity. Smaller sample size is another limitation of the study. Further studies are required with a larger sample size to identify the levels of this biomarker in predicting disease outcome.

## Conclusions

The salivary sialic acid level was more among beedi rollers than individuals with no tobacco exposure, even though the difference was not statistically significant. The results showed elevated levels of salivary sialic acid in smokeless tobacco users followed by tobacco smokers. This may be an indication that smokeless tobacco use has harmful eﬀects similar to or more than tobacco smoking since salivary sialic acid levels in smokeless tobacco users were higher than those in smokers.

Therefore, a saliva-based test could prove useful for early detection and monitoring of health eﬀects of these habits since saliva is a readily available biomaterial. And it can be made useful in community screening programs and it will be useful in imparting education to the public regarding health hazards caused by tobacco Thus, salivary sialic acid may be used as a useful marker for estimating the ill effects of tobacco exposure. Education should be imparted to beedi rollers regarding health hazards caused by tobacco. More studies should be conducted among beedi rollers as they are exposed to tobacco on a daily basis which may lead to many serious health issues.
